# Investigation of Cutting Temperature during Turning Inconel 718 with (Ti,Al)N PVD Coated Cemented Carbide Tools

**DOI:** 10.3390/ma11081281

**Published:** 2018-07-25

**Authors:** Jinfu Zhao, Zhanqiang Liu, Qi Shen, Bing Wang, Qingqing Wang

**Affiliations:** 1 School of Mechanical Engineering, Shandong University, Key Laboratory of High Efficiency and Clean Mechanical Manufacture of MOE, Jinan 250061, China; sduzhaojinfu@gmail.com (J.Z.); sdushenqi@gmail.com (Q.S.); sduwangbing@gmail.com (B.W.); sduwangqingqing@gmail.com (Q.W.); 2 Key National Demonstration Center for Experimental Mechanical Engineering Education, Jinan 250061, China

**Keywords:** PVD Ti_0.41_Al_0.59_N/Ti_0.55_Al_0.45_N coating, cutting temperature, Inconel 718

## Abstract

Physical Vapor Deposition (PVD) Ti_1−*x*_Al*_x_*N coated cemented carbide tools are commonly used to cut difficult-to-machine super alloy of Inconel 718. The Al concentration *x* of Ti_1−*x*_Al*_x_*N coating can affect the coating microstructure, mechanical and thermo-physical properties of Ti_1−*x*_Al*_x_*N coating, which affects the cutting temperature in the machining process. Cutting temperature has great influence on the tool life and the machined surface quality. In this study, the influences of PVD (Ti,Al)N coated cemented carbide tools on the cutting temperature were analyzed. Firstly, the microstructures of PVD Ti_0.41_Al_0.59_N and Ti_0.55_Al_0.45_N coatings were inspected. The increase of Al concentration *x* enhanced the crystallinity of PVD Ti_1−*x*_Al*_x_*N coatings without epitaxy growth of TiAlN crystals. Secondly, the mechanical and thermo-physical properties of PVD Ti_0.41_Al_0.59_N and Ti_0.55_Al_0.45_N coated tools were analyzed. The pinning effects of coating increased with the increasing of Al concentration *x*, which can decrease the friction coefficient between the PVD Ti_1−*x*_Al*_x_*N coated cemented carbide tools and the Inconel 718 material. The coating hardness and thermal conductivity of Ti_1−*x*_Al*_x_*N coatings increased with the increase of Al concentration *x*. Thirdly, the influences of PVD Ti_1−*x*_Al*_x_*N coated tools on the cutting temperature in turning Inconel 718 were analyzed by mathematical analysis modelling and Lagrange simulation methods. Compared with the uncoated tools, PVD Ti_0.41_Al_0.59_N coated tools decreased the heat generation as well as the tool temperature to reduce the thermal stress generated within the tools. Lastly, the influences of Ti_1−*x*_Al*_x_*N coatings on surface morphologies of the tool rake faces were analyzed. The conclusions can reveal the influences of PVD Ti_1−*x*_Al*_x_*N coatings on cutting temperature, which can provide guidance in the proper choice of Al concentration *x* for PVD Ti_1−*x*_Al*_x_*N coated tools in turning Inconel 718.

## 1. Introduction

Inconel 718 has been widely used in aeronautical applications due to its high temperature strength and high corrosion resistance. However, great heat is generated in the cutting process of Inconel 718 by cutting tools due to its low thermal conductivity and high hardness. The heat can decrease the tool life and impair the machined surface quality of a workpiece [1,2,3,4,5]. Coating technology was commonly used to protect the tool substrate, which was an effective way to increase the tool life [6,7]. At present, coated cemented carbide tools account for 80% of total tool production [8]. Physical Vapor Deposition (PVD) Ti_1−*x*_Al*_x_*N coatings were widely used in machining Inconel 718 alloys due to the high hardness [9], excellent wear resistance [10,11] and super heat stability [12] of the coatings. The thermo-physical properties of PVD Ti_1−*x*_Al*_x_*N coating were closely associated with the Al concentration *x* [13,14,15,16,17]. It is necessary to study the influences of Al concentration on the microstructure, mechanical properties and thermo-physical properties of PVD Ti_1−*x*_Al*_x_*N coatings. This can provide some guidance when choosing proper Al concentration *x* of PVD Ti_1−*x*_Al*_x_*N coated tools in turning Inconel 718.

Researchers commonly carried out experimental observation to analyze the microstructures, mechanical properties and thermo-physical properties of Ti_1−*x*_Al*_x_*N coatings. Hultman [15] found that PVD Ti_1−*x*_Al*_x_*N coating was a combination of the mostly metallic character of cubic TiN with the semi-conducting behavior of hexagonal AlN. The Al was insoluble in TiN, and Ti was insoluble in AlN. The PVD deposition conditions were far from the thermodynamic equilibrium that was required to synthesize the supersaturated metastable Ti_1−*x*_Al*_x_*N coatings. Wahlström et al. [16] analyzed the microstructures of polycrystalline Ti_1−*x*_Al*_x_*N alloy coatings, which were made by ultra-high-vacuum dual-target magnetron sputtering technologies. They found that coatings with an AlN concentration *x* ≤ 0.40 were single-phase with a face-centered cubic structure of Ti_1−*x*_Al*_x_*N coating. The interplanar distance and intergranular void density decreased with the increase of AlN concentration *x*. As detected by selected-area electron diffraction (SAED), the coatings with an AlN concentration 0.4 < *x* ≤ 0.9 consisted of wurtzite-structure AlN-rich grains and face-centered cubic structure Al depleted Ti_1−*x*_Al*_x_*N grains. Plan-view transmission electron microscopy (TEM) also revealed a dramatic decrease in the average grain size from 65 nm to 30 nm and an increase in the intergranular void density to accompany the phase separation. Yoon et al. [17] researched the influences of Al concentration *x* on the microstructure and mechanical properties of WC-Ti_1−*x*_Al*_x_*N super hard composite coatings. With the increase of Al concentration, the crystal grain interfaces of WC-Ti_0.47_Al_0.53_N coatings showed a complete nano-crystalline structure with a grain size of 10 nm. The highest hardness value of WC-Ti_1−*x*_Al*_x_*N coatings was obtained from the WC-Ti_0.43_Al_0.57_N nano-composite coating. Paldey et al. [13] found that the hardness, oxidation resistance and thermal conductivity of Ti_1−*x*_Al*_x_*N coating rose with the increase of the Al atom replacing Ti atom within the TiAlN cubic cell. Ding et al. [14] adopted time domain reflectometry to measure the variation curve of the thermal conductivity of PVD Ti_1−*x*_Al*_x_*N coating with the increase of Al concentration *x* at room temperature. They found that the thermal conductivity of PVD Ti_1−*x*_Al*_x_*N coating decreased with the increase of Al concentration *x* (*x* < 0.42), but the thermal conductivity of PVD Ti_1−*x*_Al*_x_*N coating increased with the increase of Al concentration *x* (*x* > 0.42). The thermal conductivity 4.63 W/(m·K) of PVD Ti_0.58_Al_0.42_N coating was the lowest value among the PVD Ti_1−*x*_Al*_x_*N coatings. The research [14] can offer evidence to determine the thermal conductivities of PVD Ti_0.41_Al_0.59_N and Ti_0.55_Al_0.45_N coatings in the current study. The research showed that the coating microstructures were closely associated with the thermal conductivities of PVD Ti_1−*x*_Al*_x_*N coatings. Barsoum et al. [18] measured the thermal conductivity of Ti_4_AlN_2.9_ coating made by the reactive hot isostatic pressing method. The thermal conductivity of Ti_4_AlN_2.9_ was 12 W/(m·K), which increased with the temperature to 20 W/(m·K) (1300 K). Rachbauer et al. [19] found that the thermal conductivity of Ti_1−*x*_Al*_x_*N coating initially increased, then decreased with increasing temperature. The temperature dependent thermo-physical properties of Ti_1−*x*_Al*_x_*N coatings were closely associated with the crystallinity states of coating and the scattering effects of the grain boundaries.

Researchers commonly utilized the mathematical analysis method [20], experimental test method [13,21] and numerical simulation method [22,23] to analyze the influences of tool coating on the cutting temperature. Du et al. [20] used the single domain and multi domain boundary element methods to calculate the temperature distribution within the coated tool. The internal temperature distribution within the tools can be influenced by the coating material and coating thickness. The calculated temperature values of the tool substrate for the TiN coated tool were slightly lower than those of the corresponding point within the uncoated tools. Paldey et al. [13] indicated that the thermal barrier effect of Ti_1−*x*_Al*_x_*N coating was due to its lower thermal conductivity than that of the substrate during a high speed dry machining process. Müller et al. [21] applied a two-color pyrometer to measure the cutting temperature in machining 42CrMo4V by uncoated tools, TiN and TiAlN coated tools. For the cutting speed 150 m/min, feed 0.12 mm/rev and cutting depth 2.5 mm, the maximum temperature of chip formed by uncoated tool was about 480 °C. The maximum temperatures in chips formed by TiN and TiAlN coated tools were about 520 °C and 500 °C, respectively. Davoudinejad et al. [22] proposed 3D finite element modeling of micro end-milling Al6082-T6 to analyze the temperature distribution under different cutting conditions. The simulation results were verified by infrared thermal camera. Grzesik [23] found that the microstructure of coating can be optimized to reduce the friction coefficient between the coated tools and workpiece materials. The microstructure of coatings can be optimized to decrease the heat generated in the cutting zone. For cutting easy-to-machine materials with coated tools, the generated heat can be easily dissipated into the chip by the coatings with lower thermal conductivity. For cutting the difficult-to-machine materials with coated tools, the generated heat can be quickly dissipated into the tool substrate by the coatings with higher thermal conductivity.

However, the influences of PVD Ti_1−*x*_Al*_x_*N coated tools with different Al concentration *x* on the cutting temperature in machining Inconel 718 were not clear. In this study, Ti_0.55_Al_0.45_N and Ti_0.41_Al_0.59_N coatings with the coating thickness 2 μm were deposited on WC-Co cemented carbide substrate. The deposition technique was arc ion plating deposition technology. The microstructure and grain orientation of the two coatings were observed by high resolution transmission electron microscopy/focused ion beam (HRTEM/FIB) techniques. The mechanical properties of the two coatings were researched by measuring the coating hardness, critical loads between the coating and substrate, and the friction coefficient between the coating and Inconel 718. The influences of Al concentration *x* on the thermal conductivity of PVD Ti_1−*x*_Al*_x_*N coatings were analyzed with the finite element method. The cutting temperatures of PVD Ti_1−*x*_Al*_x_*N coated tools were measured with the buried thermocouples. The influences of PVD Ti_1−*x*_Al*_x_*N coating on heat generation in the cutting zone were analyzed with mathematical analysis models.

## 2. Mathematical Analysis Model of Cutting Temperature in Turning Inconel 718 by PVD Ti_1−*x*_Al*_x_*N Coated Tools

**Figure 1:**
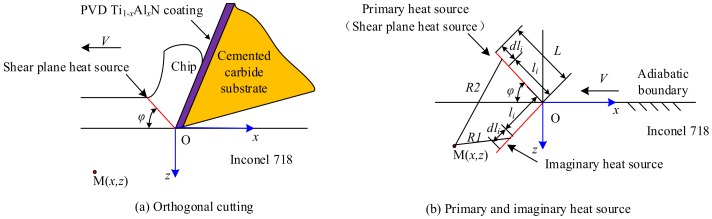
Mathematical analysis model of the primary and imaginary shear heat sources.

The orthogonal mathematical analysis models proposed by Komanduri-Hou [24,25] were used to investigate the cutting temperature of the primary cutting zone in turning Inconel 718 with PVD Ti_1−*x*_Al*_x_*N coated cemented carbide tools. Figure 1 shows the mathematical analysis model of the primary and imaginary shear heat sources in orthogonal cutting of Inconel 718 by PVD Ti_1−*x*_Al*_x_*N coated tools.

The separating point of the tool tip and workpiece is assumed as the coordinate origin *O* as shown in Figure 1. The direction parallel to the cutting speed is assumed as the direction of the *x* axis, and the direction perpendicular to the cutting speed is assumed as the direction of the *z* axis. The two-dimensional planar coordinate *xOz* is established. *φ* is the shear angle. *V* is the cutting speed. *L* is the length of shear heat source. *R*_1_ and *R*_2_ are the polar coordinates of point M(*x*, *z*). The imaginary heat source was introduced into the model to compensate for the heat loss due to the assumption of the semi-infinite medium for the workpiece [24,25].

The temperature rise at any point M(*x*, *z*) was due to the combined effects of the primary and imaginary shear heat sources. Each of these heat sources can be considered as a combination of numerous infinitesimal segments *dl_i_*, with each again as an infinitely long moving line heat source. *l_i_* is the location of the differential small segment of the shear band heat source *dl_i_* relative to the upper end of it and along its width. *K*_0_ is the modified Bessel function of second kind of order zero. The temperature of point M(*x*, *z*) within the workpiece induced by the primary and imaginary shear heat sources can be calculated by Equation (1):

(1) $$\begin{array}{l} T_{M(x,z)}^{shear}=T_{\mathrm{primary}}+T_{\mathrm{imaginary}}\\ =B\frac{q_{\mathrm{shear}}}{2\pi \lambda }\int_{l_{i}=0}^{L}e^{-\left(x-l_{i}\cdot cos(\varphi )\right)\cdot V/2\alpha }\cdot \\ \text{\{}K_{0}\left[\frac{V}{2\alpha }\sqrt{{\left(x-l_{i}\cos\left(\varphi \right)\right)}^{2}+{\left(z-l_{i}\sin\left(\varphi \right)\right)}^{2}}\right]+\\ K_{0}\left[\frac{V}{2\alpha }\sqrt{{\left(x-l_{i}\cos\left(\varphi \right)\right)}^{2}+{\left(z+l_{i}\sin\left(\varphi \right)\right)}^{2}}\right]\}dl_{i} \end{array}$$

where *T*_primary_ and *T*_imaginary_ are temperatures generated by the primary and imaginary shear heat sources, respectively. The temperatures can be calculated with Equations (2) and (3). *B* is the coefficient under specific cutting parameters in machining Inconel 718, which can be calculated with Equation (4). *q*_shear_ is the heat liberation intensity of a moving shear plane heat source, which can be calculated with Equation (5). *λ* is the thermal conductivity of Inconel 718. *α* is the thermal diffusivity of Inconel 718. *K*_0_ can be calculated with Equation (6).

(2) $$T_{\mathrm{primary}}=B\frac{q_{\mathrm{shear}}}{2\pi \lambda }\int_{l_{i}=0}^{L}e^{{}^{{}^{-\left(x-l_{i}\cdot cos(\varphi )\right)\cdot V/2\alpha }}}\times K_{0}\frac{V}{2\alpha }\sqrt{{\left(x-l_{i}\cos\left(\varphi \right)\right)}^{2}+{\left(z-l_{i}\sin\left(\varphi \right)\right)}^{2}}dl_{i}$$

(3) $$T_{\mathrm{imaginary}}=B\frac{q_{\mathrm{shear}}}{2\pi \lambda }\int_{l_{i}=0}^{L}e^{{}^{{}^{{}^{-\left(x-l_{i}\cdot cos(\varphi )\right)\cdot V/2\alpha }}}}\times K_{0}\frac{V}{2\alpha }\sqrt{{\left(x-l_{i}\cos\left(\varphi \right)\right)}^{2}+{\left(z+l_{i}\sin\left(\varphi \right)\right)}^{2}}dl_{i}$$

(4) $$B=0.60361\times N_{\mathrm{th}}^{-0.37101}=0.60361\times {\left(\frac{t_{c}\times V}{\alpha }\right)}^{-0.37101}$$

(5) $$q_{shear}=\frac{\left(F_{c}\cos(\varphi )-F_{t}\sin(\varphi ))\cdot V\cdot \cos(\gamma_{0}\right)}{1000t_{c}\cdot w\cdot \csc(\varphi )\cdot \cos(\varphi -\gamma_{0})}$$

where *F_c_* is the tangential force, *F_t_* is the radial force, *γ*_0_ is the rake angle, *t_c_* is the undeformed chip thickness, and *w* is the width of shear plane heat source.

(6) $$K_{0}=\frac{1}{2}\int_{0}^{\infty }\frac{d\omega }{\omega }e^{\left(-\omega -\frac{u^{2}}{4\omega }\right)}$$

where *ω* is the variable of cutting time, which can be calculated by Equation (7), and *u* is the integral variable of shear band length, which can be calculated by Equation (8).

(7) $$\omega =\frac{V^{2}t}{4\alpha }$$

(8) $$u=\frac{V}{2\alpha \cdot l_{i}\cdot \cos(\varphi )}$$

## 3. Experimental Procedure

### 3.1. Characterization of Surface and Microstructure for PVD Ti_1−x_Al_x_N Coatings

The surface roughness of PVD Ti_1−*x*_Al*_x_*N coating was obtained with a laser confocal microscope VK-H1XMC (Keyence, Osaka, Japan). The tool rake face was set perpendicular to the measuring lens. The amplification of the lens used was 10×, which could cover the valid area of the tool rake face.

PVD Ti_1−*x*_Al*_x_*N coatings were characterized structurally by X-ray diffraction (XRD, Shimadzu, Kanagawa, Japan) and HRTEM/FIB techniques. For the XRD analyses, a wide-range goniometer with a proportional-counter detector was used, with a 2*θ* accuracy of 0.0001°. Non-monochromatic Cu radiation was used and K_α_ peaks were numerically stripped from the spectra using an EVA and TOPAS 4.2 software package. The pole figures were obtained using a *X* ray diffractometer with the type of D8 advance. The FIB preparation was performed with a FEI Helios 600 Dual Beam, consisting of a liquid gallium ion source operating at 30 kV for sample milling and a field emission electron source operating at 5 kV for secondary electron imaging. The thin lamellae were imaged with a FEI S-TWIN TECNAI G^2^ F20-TEM (FEI, Hillsboro, OR, America) operating at 200 kV, to carry out experiments with HRTEM, selected area electron diffraction (SAED), and dark-field imaging.

The cross sections of PVD Ti_1−*x*_Al*_x_*N coatings were observed with the field emission scanning electron microscope JSM-7610F (JEOL, Tokyo, Japan). The accelerating voltage was 10 kV. The detailed cross sections of PVD Ti_1−*x*_Al*_x_*N coatings were observed at the magnification 20,000×.

### 3.2. Characterization of Mechanical Properties for PVD Ti_1−x_Al_x_N Coated Tools

As referred to in [14,15], the hardness of PVD Ti_1−*x*_Al*_x_*N coating was measured with the Vickers hardness tester FM-800. The applied method was the indentation method. It was noted that the indentation depth of the indenter cannot exceed 10–15% of the coating thickness to assure the validity of the measured results. The applied load of the indenter was 50 g.

The Anton Par Revetest was used to apply the scratching tests to obtain the critical loads between the PVD Ti_1−*x*_Al*_x_*N coating and carbide substrate. The tool rake faces were set perpendicular to the Rockwell diamond indenter C, then the tools were fixed. The cone angle of the Rockwell diamond indenter C was 120°, the curvature radius of which was 200 μm. The applied load was in the range of 0–60 N for the Rockwell diamond indenter C. The scratching tests met the requirements of ASTMC1624-05 standard [26]. The scratching speed was 10 mm/min, and the loading rate was 100 N/min. The rupturing sound of the coating and the three-dimensional topography of the scratch were used to find the critical loads between the PVD Ti_1−*x*_Al*_x_*N coating and carbide substrate. The detailed measuring process can be referred to the research [27].

The friction coefficient between the PVD Ti_1−*x*_Al*_x_*N coated tool and workpiece material Inconel 718 was measured with pin-on-disc testing experiments. The experimental setup was with a type of UMT-TriboLab (Bruker, Billerica, MA, USA). The Inconel 718 was made as the disc with the diameter of 80 mm. The loads applied on the tool were a constant of 5 N at the room temperature.

### 3.3. Cutting Experiment Procedure

**Figure 2:**
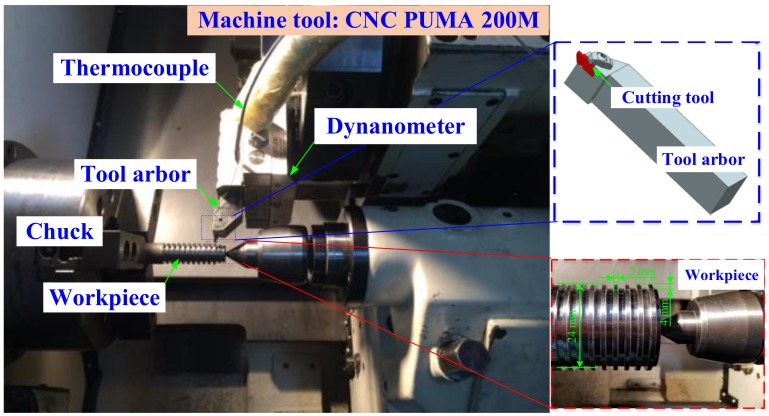
Experimental setup of orthogonal cutting Inconel 718 by PVD Ti_1−*x*_Al*_x_*N coated tools without cutting lubricant, the illustration shows the coordinate axis of the machine tool.

The experimental setup of orthogonal cutting Inconel 718 by PVD Ti_1−*x*_Al*_x_*N coated tools without cutting lubricant is shown in Figure 2. The used machine tool was CNC PUMA 200 M. The PVD Ti_1−*x*_Al*_x_*N coated cemented carbide tools with the type NG3125R KC5025 (PVD Ti_0.55_Al_0.45_N coated tool) and KC5010 (PVD Ti_0.41_Al_0.59_N coated tool) were obtained from the KENNAMETAL company. As referred to the methodologies of research [15,17], the thickness of the PVD Ti_1−*x*_Al*_x_*N coatings were measured as 2 μm from the cross-sectional views of the coated tools using scanning electron microscopy (SEM) techniques. PVD Ti_1−*x*_Al*_x_*N coated tools were compared with the uncoated cemented carbide tools with the NG3125R K313. The three type cutting tools had the same rake angle *γ*_0_ 0°, relief angle *β* 11° and geometric dimensions. PVD Ti_0.55_Al_0.45_N and Ti_0.41_Al_0.59_N coated tools had the same substrate material as the uncoated tool. The cutting tool arbor with the type of NSR 3232P3 was shown in the illustration in the Figure 2. The workpiece of Inconel 718 was a cylindrical bar with the diameter of *φ*24 mm. The ring grooves had been machined on the cylindrical bar. The depth of the ring grooves was 4 mm. The distance was 2 mm between two approached ring grooves.

**Figure 3:**
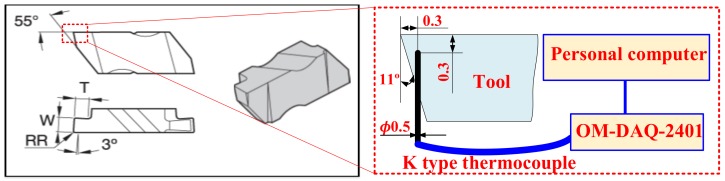
Schematic of temperature measurement with buried K-type thermocouple.

The applied cutting parameters of PVD Ti_1−*x*_Al*_x_*N coated tool and uncoated tool were the same. The cutting speed used was 20 m/min. The feed used was 0.025, 0.05 and 0.075 mm/rev, respectively. As shown in Figure 2, the cutting forces were measured with the Type 9129A of 3-Component Measuring System. The cutting temperature of tools was measured with the buried K type thermocouple, which was combined with a multiple channel USB data acquisition module OM-DAQ-2401. The schematic of temperature measurement was plotted in Figure 3. As shown in Figure 3, the diameter of the K-type thermocouple was *φ*0.5 mm. The diameter of the un-displayed drilled hole was *φ*0.75 mm, which was made by electric discharge machining. The response time of the multiple channel USB data acquisition module OM-DAQ-2401 was 2 ms. The collected electric signals were transferred into the personal computer. Thus, the temperature profiles of tools were obtained accurately during the Inconel 718 turning process.

## 4. Finite Element Simulation of Cutting Temperature in Turning Inconel 718 with PVD Ti_1−*x*_Al*_x_*N Coated Tools

**Figure 4:**
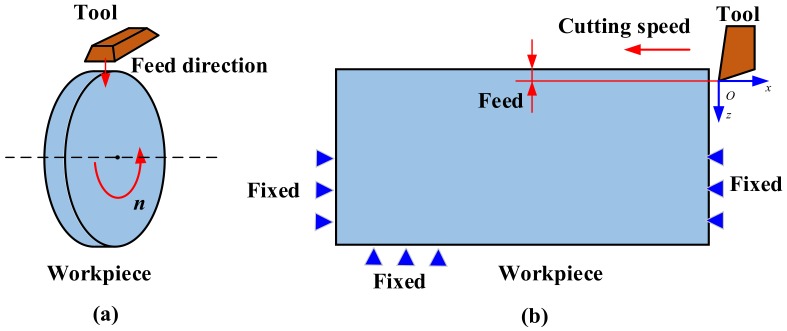
Schematic diagram of experimental set-up and the related simulation models. (**a**) Experimental model; (**b**) simulation model.

The schematic diagram of the experimental setup of the orthogonal cutting of Inconel 718 by PVD Ti_1−*x*_Al*_x_*N coated tools is plotted in Figure 4a and the schematic diagram of the related simulation model is plotted in Figure 4b. As in [28,29,30,31], the finite element simulation models were established by AdvantEdge software V5.0 without lubricants. As shown in Figure 4b, the cutting tool tip was set at a distance away from the top surface of workpiece. The distance was equal to the feed value. The workpiece was fixed and the tool moved in the cutting direction. The moving speed was equal to the cutting speed value. The feed and cutting speed values were set as shown in Table 1. The influences of PVD Ti_0.41_Al_0.59_N and Ti_0.55_Al_0.45_N coated tools on the cutting temperature in turning Inconel 718 were analyzed by applying the coatings on the cutting tool. The specific thermo-physical properties of PVD Ti_0.41_Al_0.59_N and Ti_0.55_Al_0.45_N coatings and the carbide substrate materials were assumed to be the values shown in Table 2. The initial temperature was set to be 20 °C, the same as room temperature. The friction coefficient between PVD Ti_1−*x*_Al*_x_*N coated tools and Inconel 718 was assumed to be the values shown in Table 1.

**Figure 5:**
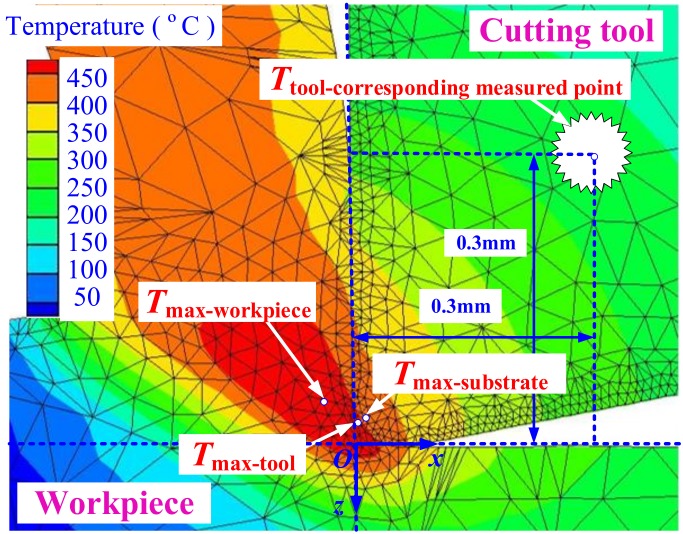
Two-dimensional finite element simulation model of cutting temperature field in turning Inconel 718 with PVD Ti_0.41_Al_0.59_N coated tool at feed 0.05 mm/rev and cutting speed 20 m/min.

As shown in Figure 5, the two-dimensional finite element simulation model of cutting temperature field in turning Inconel 718 by a PVD Ti_0.41_Al_0.59_N coated tool at feed 0.05 mm/rev and cutting speed 20 m/min were given. *T*_max-workpiece_ is the maximum temperature of workpiece. *T*_max-tool_ is the maximum temperature of tool. *T*_max-substrate_ is the maximum temperature of tool substrate. *T*_tool-corresponding measured point_ is the temperature at the measured point of the buried K type thermocouple.

## 5. Results and Discussion

### 5.1. Microstructure of PVD Ti_1−x_Al_x_N Coatings

**Figure 6:**
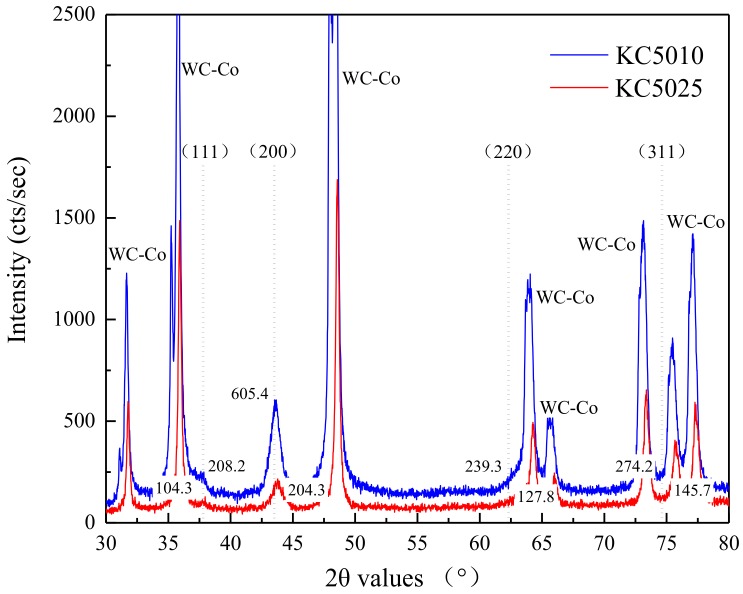
X-ray diffraction (XRD) patterns from PVD Ti_0.41_Al_0.59_N (KC5010) and Ti_0.55_Al_0.45_N (KC5025) coatings.

Figure 6 illustrates the XRD patterns from PVD Ti_0.41_Al_0.59_N (KC5010) and Ti_0.55_Al_0.45_N (KC5025) coatings. PVD Ti_0.41_Al_0.59_N (KC5010) and Ti_0.55_Al_0.45_N (KC5025) coatings were all face-centered cubic structure Ti_(1*−x*)_Al*_x_*N with cubic lattice. The space groups of two type PVD Ti_1−*x*_Al*_x_*N coatings were the same as Pm-3m (211). For the thin coating thickness and high penetrating capacity of Cu-Ka radiation used in the XRD experiment, the diffraction peaks for the WC-Co carbide substrate were identified clearly. In this research, the crystals of PVD Ti_1−*x*_Al*_x_*N coatings grew in preferred orientations (111) and (200). The grain preferred orientations (111) and (200) for the PVD Ti_1−*x*_Al*_x_*N coating became more evident with the increase of Al concentration.

**Figure 7:**
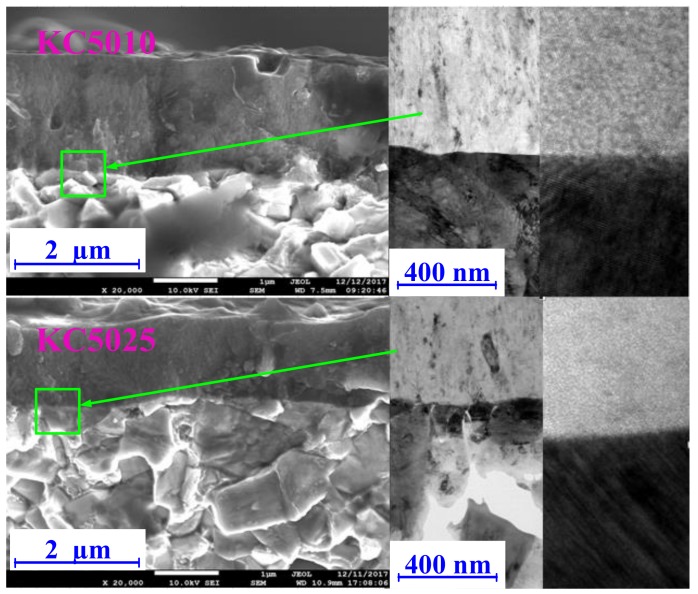
Cross-sectional scanning electron microscopy (SEM) topographies and plan-view high resolution transmission electron microscopy (HRTEM) images from the interface areas between the PVD Ti_1−*x*_Al*_x_*N coating and carbide substrate for Ti_0.41_Al_0.59_N (KC5010) coated tool and Ti_0.55_Al_0.45_N (KC5025) coated tool.

As shown in Figure 7, the interface areas between the PVD Ti_1−*x*_Al*_x_*N coating and carbide substrate were observed with SEM and HRTEM. Differences between cross-sectional SEM topographies were not evident for the Ti_0.41_Al_0.59_N (KC5010) coated tool and Ti_0.55_Al_0.45_N (KC5025) coated tool. Plan-view HRTEM images showed that the disordering areas and lattice distortions existed around the interface areas between the PVD Ti_1−*x*_Al*_x_*N coating and carbide substrate. The lattice fringe orientations of two PVD Ti_1−*x*_Al*_x_*N coatings were not consistent with the lattice fringe orientations of the WC phase and Co phase in the WC-Co carbide substrate. It was illustrated that the epitaxy growth of TiAlN crystal did not exist at the interface between the PVD Ti_1−*x*_Al*_x_*N coating and carbide substrate. The epitaxy growth of TiAlN crystal did not exist in the physical vapor deposition process. The deposition temperature of physical vapor deposition process was lower than that of the chemical vapor deposition process. Thus, the re-nucleation and growth of TiAlN crystals occurred independently in the physical vapor deposition process of PVD Ti_1−*x*_Al*_x_*N coatings without the influences of the WC phase and Co phase.

**Figure 8:**
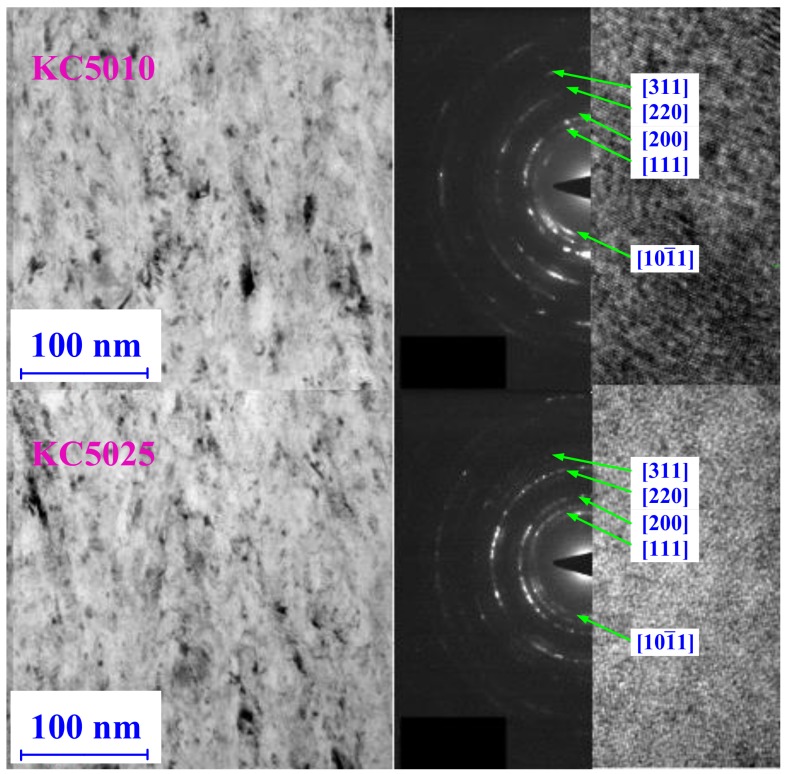
Plan-view HRTEM images and corresponding selected-area electron diffraction (SAED) patterns from PVD Ti_0.41_Al_0.59_N (KC5010) coating and PVD Ti_0.55_Al_0.45_N (KC5025) coating.

Attention was paid to the internal microstructures of PVD Ti_1−*x*_Al*_x_*N coatings. As shown in Figure 8, the internal microstructures of PVD Ti_0.41_Al_0.59_N (KC5010) and Ti_0.55_Al_0.45_N (KC5025) coatings were observed by HRTEM and SAED. The crystallizations of Ti_0.41_Al_0.59_N (KC5010) and Ti_0.55_Al_0.45_N (KC5025) coatings were all nano-crystalline. The corresponding SAED patterns showed that the continuity of diffraction facula for Ti_0.41_Al_0.59_N (KC5010) coating was not as good as that of Ti_0.55_Al_0.45_N (KC5025) coating. As referred to in [16,17], this can be explained by the nano-crystalline of Ti_0.41_Al_0.59_N (KC5010) coating being larger than that of Ti_0.55_Al_0.45_N (KC5025) coating. The crystallization states of Ti_0.41_Al_0.59_N (KC5010) coating were better than that of Ti_0.55_Al_0.45_N (KC5025) coating.

### 5.2. Mechanical and Thermo-Physical Properties of PVD Ti_1−x_Al_x_N Coatings

The mechanical properties of PVD Ti_1−*x*_Al*_x_*N coatings were listed in Table 1. Each experiment was conducted more than three times to obtain average values. As referred to in [32,33], the replacement of Ti atoms by Al atoms induced the lattice distortion of Ti_1−*x*_Al*_x_*N coating. The lattice distortion induced internal stress within the Ti_1−*x*_Al*_x_*N coatings. The increase of Al concentration increased the pinning effect in the PVD Ti_1−*x*_Al*_x_*N coating. The pinning effects prevented the dislocation movement of Ti_1−*x*_Al*_x_*N crystals. The friction coefficients between the Ti_1−*x*_Al*_x_*N coating and Inconel 718 decreased slightly [34]. Thus, the friction forces between the PVD Ti_0.41_Al_0.59_N (KC5010) coated tools and Inconel 718 decreased slightly.

The temperature-dependent mechanical and thermo-physical properties of tungsten-based cemented carbide are referenced to Akbar et al. [29]. Barsoum et al. [18] and Finkel et al. [34] found that the thermal conductivity of Ti_4_AlN_3_ increased linearly with temperature. Ti_4_AlN_3_ is another expression of (Ti,Al)N material, defined by the Ti/Al atomic ratio. Akbar et al. [29] summarized the research results of Barsoum et al. [18] and Finkel et al. [34] and obtained the temperature-dependent mechanical and thermo-physical properties of Ti_1−*x*_Al*_x_*N coating. To investigate the influences of Ti_1−*x*_Al*_x_*N coating on the cutting temperature in turning Inconel 718, the temperature-dependent mechanical and thermo-physical properties of Ti_0.41_Al_0.59_N coating were assumed to be the same as the research results of Akbar et al. [29]. According to the research of Ding et al. [14], the thermal conductivity of PVD Ti_0.55_Al_0.45_N coating is referred to as 4.6 W/(m·K) at room temperature. The thermal conductivity of PVD Ti_0.41_Al_0.59_N coating was inferred as 6.6 W/(m·K) at room temperature. Thus, the thermal conductivity of PVD Ti_0.55_Al_0.45_N coating was assumed to be less than that of PVD Ti_0.41_Al_0.59_N coating about 2 W/(m·K) with the variation of temperature. The temperature-dependent mechanical and thermo-physical properties of Ti_0.41_Al_0.59_N coating, Ti_0.55_Al_0.45_N coating and tungsten-based cemented carbide are given as Table 2.

### 5.3. Influences of PVD Ti_1−x_Al_x_N Coated Tools on Cutting Temperature

**Figure 9:**
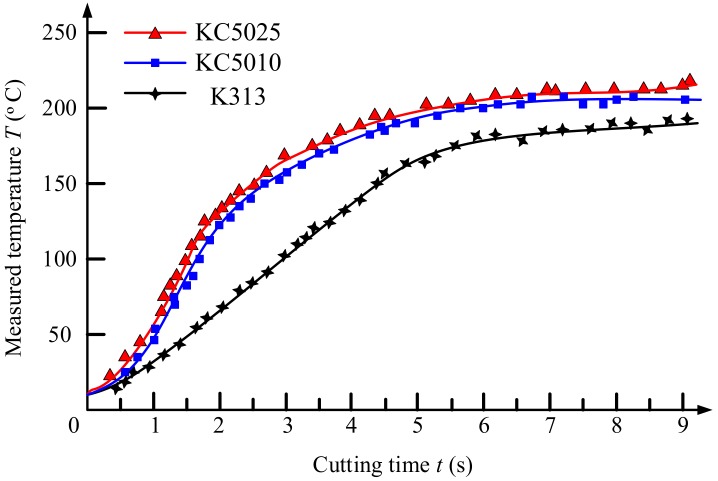
Cutting temperature profiles with variations of cutting time of PVD Ti_0.41_Al_0.59_N coated tool (KC5010), Ti_0.45_Al_0.55_N coated tool (KC5025) and uncoated tool (K313) measured by the buried K-type thermocouple at feed 0.05 mm/rev and cutting speed 20 m/min.

As shown in Figure 9, the cutting temperature profiles with variations of cutting time of Ti_0.41_Al_0.59_N coated tool (KC5010), Ti_0.55_Al_0.45_N coated tool (KC5025), uncoated tool (K313) were measured by the buried K-type thermocouple at feed 0.05 mm/rev and cutting speed 20 m/min. Compared with the uncoated tools, the existence of PVD Ti_1−*x*_Al*_x_*N coating increased the measured cutting temperature. As referred to in the research of Grezsik [23], the heat generated in the cutting zone can be prevented from dissipating from the tool body into the environment quickly by PVD Ti_1−*x*_Al*_x_*N coated tools. Thus, the heat that accumulated within the tool body increased the measured temperature. The measured temperatures of PVD Ti_0.55_Al_0.45_N coated tool were higher than those of the PVD Ti_0.41_Al_0.59_N coated tool. This was associated with the thermal conductivity of Ti_(1*−x*)_Al*_x_*N coating being increased with the increase of Al concentration [18,19]. The Al concentration of Ti_0.41_Al_0.59_N coating was higher than that of the Ti_0.55_Al_0.45_N coating. Thus, the thermal conductivity of Ti_0.41_Al_0.59_N coating was higher than Ti_0.55_Al_0.45_N coating as referred to in [14,18,19]. Compared with the PVD Ti_0.55_Al_0.45_N coated tools, the heat generated can be dissipated quickly from the tool body into the environment and thus decrease the measured temperature for PVD Ti_0.41_Al_0.59_N coated tools.

**Figure 10:**
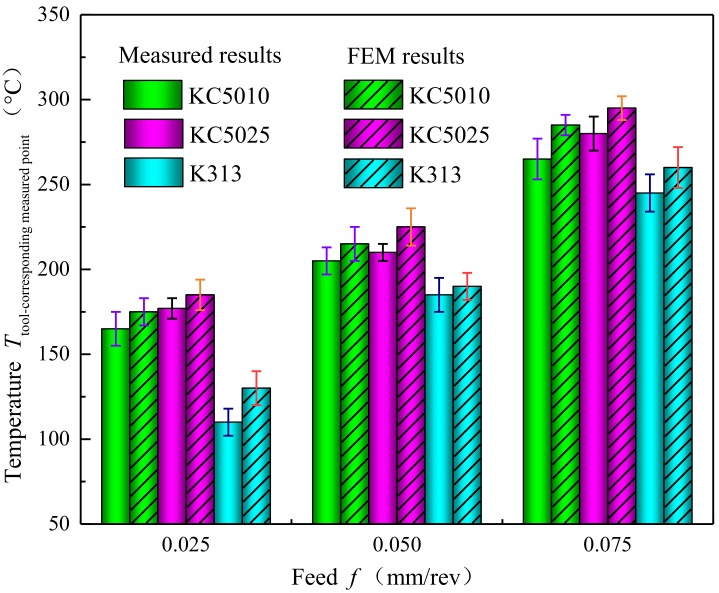
Comparisons between the actual temperature measured by the buried K-type thermocouple and the corresponding finite element simulation temperature at the same point within the substrate of Ti_0.41_Al_0.59_N coated tool (KC5010), Ti_0.45_Al_0.55_N coated tool (KC5025) and uncoated tool (K313) with variations of feed at cutting speed 20 mm/min.

Comparisons between the actual temperature measured by the buried K-type thermocouple and the corresponding finite element simulation temperature at the same point within the substrate of tools with variations of feed are illustrated in Figure 10. The variations of finite element simulation temperature were consistent with the measured temperatures at the same point within the tool substrates. The finite element analysis model can show the temperature field in the cutting zone directly. Compared with the uncoated tools, the existence of PVD Ti_(1*−x*)_Al*_x_*N coating increased the steady temperature in turning Inconel 718. The measured temperature of PVD Ti_0.41_Al_0.59_N coated tools was lower than that of PVD Ti_0.55_Al_0.45_N coated tools. The Ti_0.41_Al_0.59_N coating with higher thermal conductivity accelerated the heat generated in the cutting zone dissipating from the tool body into the environment.

**Figure 11:**
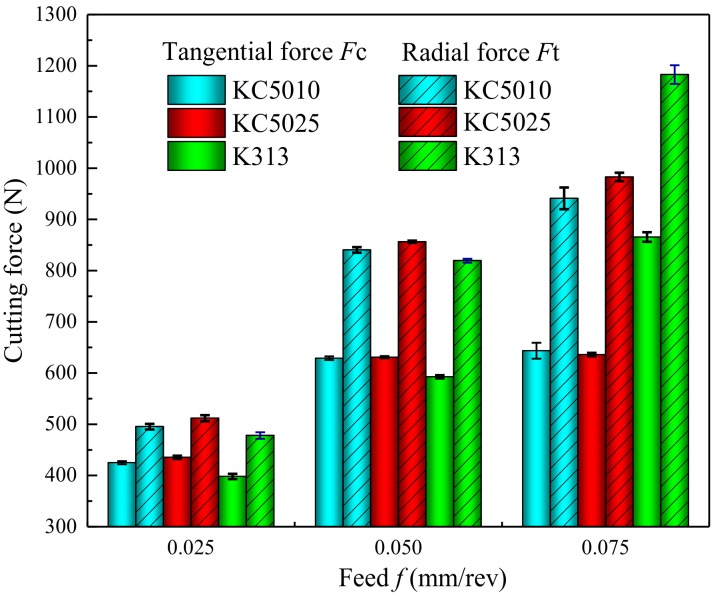
Tangential forces (*Ft*) and radial forces (*Fc*) of PVD Ti_0.41_Al_0.59_N coated tool (KC5010), Ti_0.45_Al_0.55_N coated tool (KC5025), uncoated tool (K313) with variations of feed at cutting speed of 20 mm/min.

To analyze the phenomenon, the tangential forces (*Ft*) and radial forces (*Fc*) of PVD Ti_0.41_Al_0.59_N coated tool (KC5010), PVD Ti_0.55_Al_0.45_N coated tool (KC5025), uncoated tool (K313) with variations of feed at a cutting speed 20 mm/min were obtained, as shown in Figure 11. The tangential forces (*Ft*) and radial forces (*Fc*) increased with the increase of feed. The incremental rate of radial force (*Fc*) was bigger than that of the tangential force (*Ft*). The radial forces decreased with the increase of Al concentration in PVD Ti_(1*−x*)_Al*_x_*N coating in turning Inconel 718. The tangential forces of PVD Ti_0.41_Al_0.59_N coated tools were lower than that of the PVD Ti_0.55_Al_0.45_N coated tools at low feed 0.025 mm/rev. The difference between the tangential forces of the PVD Ti_0.41_Al_0.59_N coated tool and the tangential forces of the PVD Ti_0.55_Al_0.45_N coated tool were not evident at higher feeds.

To analyze the influence of PVD Ti_(1*−x*)_Al*_x_*N coated tools on the heat generation in turning Inconel 718, the special parameters of adiabatic shear fracture in the cutting zone were calculated with the measured cutting forces, according to Equations (4)–(8). The special parameters of adiabatic shear fracture in the cutting zone for PVD Ti_0.41_Al_0.59_N coated tool (KC5010), Ti_0.55_Al_0.45_N coated tool (KC5025) and uncoated tool (K313) tools are shown in Table 3. The confidence intervals of the obtained values were 95%. According to the variation of heat liberation intensity *q*_shear_ of a moving shear plane heat source with feeds, the existence of PVD Ti_(1*−x*)_Al*_x_*N coating increased the heat generation in the cutting zone to dissipate more heat into the tool body. This was consistent with the phenomenon that the actual measured temperatures of PVD Ti_(1*−x*)_Al*_x_*N coated tool were higher than the uncoated tools. Compared with the PVD Ti_0.55_Al_0.45_N coated tools, PVD Ti_0.41_Al_0.59_N coated tools decreased the heat liberation intensity *q*_shear_ of a moving shear plane heat source and decreased the heat dissipating into the tool under the same cutting parameters. This was consistent with the phenomenon of the actual measured temperatures of the PVD Ti_0.41_Al_0.59_N coated tool being lower than that of PVD Ti_0.55_Al_0.45_N coated tools. The thermal conductivity of PVD Ti_0.41_Al_0.59_N coating was higher than that of the PVD Ti_0.55_Al_0.45_N coating [35]. PVD Ti_0.55_Al_0.45_N coated tools generated more heat in the cutting zone and dissipated more heat from the tool body into the environment. Thus, the cutting temperatures of the PVD Ti_0.41_Al_0.59_N coated tool measured by the buried K type thermocouple were lower than that of the PVD Ti_0.55_Al_0.45_N coated tool.

**Figure 12:**
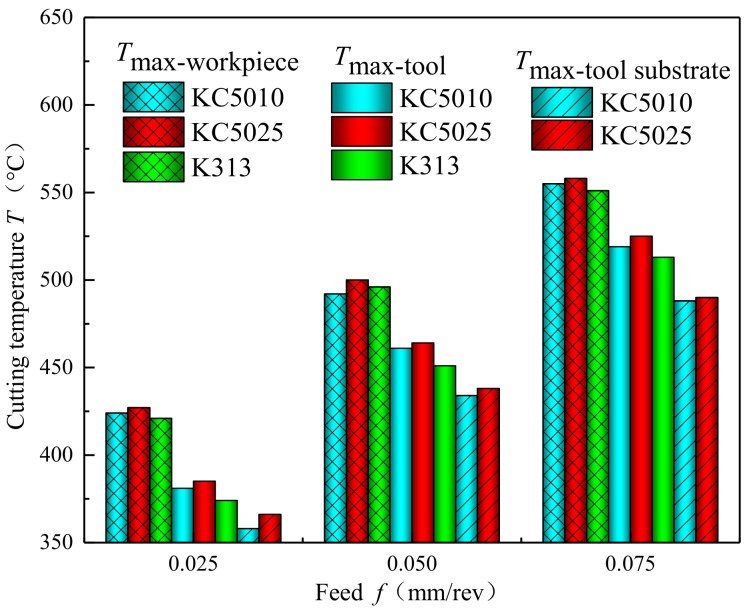
Comparisons of the maximum temperature of the workpiece (*T*_max-workpiece_), the maximum temperature of the tool (*T*_max-tool_) and the maximum temperature of the tool substrate (*T*_max-substrate_) in the finite element simulation models with variations of feed at a cutting speed of 20 m/min for Ti_0.41_Al_0.59_N coated tool (KC5010), Ti_0.45_Al_0.55_N coated tool (KC5025) and uncoated tool (K313).

As shown in Figure 12, the influences of PVD Ti_(1*−x*)_Al*_x_*N coatings on the maximum temperature of the workpiece (*T*_max-workpiece_), the maximum temperature of the tool (*T*_max-tool_) and the maximum temperature of the tool substrate (*T*_max-substrate_) in the finite element simulation models were plotted with variations of feed at a cutting speed of 20 m/min. Compared with the uncoated tool, the existence of PVD Ti_(1*−x*)_Al*_x_*N coatings increased the maximum temperature of the workpiece and increased the maximum temperature of the tool. This phenomenon was different from the former research results of Grezsik et al. [36]. Grezsik et al. [36] found that coated tools can decrease the cutting temperature during cutting 45 steel with calculation and simulation methods. Compared with 45 steel, Inconel 718 was used as the hard-to-machine material for its low thermal conductivity and the difficult deformation characteristics. The deformation of Inconel 718 can generate massive heat in the cutting zone. The generated heat cannot be quickly dissipated by chips like the 45 steels. The generated heat in the cutting zone accumulated to increase the maximum temperature of the workpiece in machining Inconel 718 by PVD Ti_(1*−x*)_Al*_x_*N coated tools.

Compared with the uncoated tools, the existence of PVD Ti_(1*−x*)_Al*_x_*N coating also increased the maximum temperature of the coated tool. Compared with the uncoated tools, PVD Ti_(1*−x*)_Al*_x_*N coated tools decreased the maximum temperature of the tool substrate to reduce the thermal stresses within the tools. This was due to the thermal barrier effects of PVD Ti_(1*−x*)_Al*_x_*N coating [23]. Compared with PVD Ti_0.55_Al_0.45_N coated tools, PVD Ti_0.41_Al_0.59_N coated tools decreased the maximum temperature of the workpiece, the maximum temperature of the tool, and the maximum temperature of the tool substrate to reduce the heat generated in the cutting zone. The heat dissipated from the cutting zone into the tool body also decreased in turning Inconel 718 by the PVD Ti_0.41_Al_0.59_N coated tool. The decreased maximum temperature of tool substrate for machining Inconel 718 by the PVD Ti_0.41_Al_0.59_N coated tool also decreased the generated thermal stresses in the tools under the same cutting parameters.

### 5.4. Influences of PVD Ti_1−x_Al_x_N Coating on the Surface Topographies of the Tool Rake Faces

**Figure 13:**
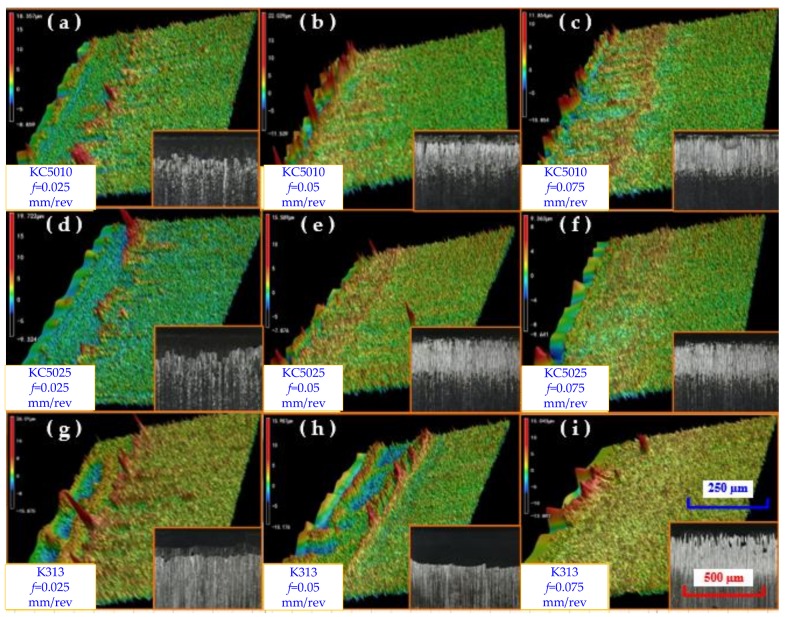
Surface topographies of the tool rake faces for PVD Ti_0.41_Al_0.59_N coated tool (KC5010), Ti_0.45_Al_0.55_N coated tool (KC5025) and uncoated tool (K313) with variations of feed at a cutting speed of 20 m/min. (**a**) KC5010, *f* = 0.025 mm/rev, (**b**) KC5010, *f* = 0.05 mm/rev, (**c**) KC5010, *f* = 0.075 mm/rev, (d) KC5025, *f* = 0.025 mm/rev, (**e**) KC5025, *f* = 0.05 mm/rev, (**f**) KC5025, *f* = 0.075 mm/rev, (**g**) K313, *f* = 0.025 mm/rev, (**h**) K313, *f* = 0.05 mm/rev, (**i**) K313, *f* = 0.075 mm/rev.

The surface topographies of the tool rake face for the Ti_0.41_Al_0.59_N coated tool (KC5010), Ti_0.45_Al_0.55_N coated tool (KC5025) and uncoated tool (K313) were characterized with a laser confocal microscope of the type VK-H1XMC. Surface topographies of the tool rake faces for Ti_0.41_Al_0.59_N coated tool (KC5010), Ti_0.45_Al_0.55_N coated tool (KC5025) and uncoated tool (K313) with variations of feed at a cutting speed of 20 m/min were obtained as shown in Figure 13a–i. It was seen that the shiny components were the Inconel 718 workpiece material adhered on the tool rake face.

The adhesion of workpiece material on the tool rake face increased with the increase of feed. The existence of PVD Ti_(1*−x*)_Al*_x_*N coating can increase the wear resistance of tools. The wear of uncoated tools (K313) were evident at the feed 0.075 mm/rev and a cutting speed of 20 m/min. The wear of uncoated tools increased the cutting forces and increased the heat liberation intensity *q*_shear_ of a moving shear plane heat source. As shown in Table 2, the heat liberation intensity *q*_shear_ of a moving shear plane heat source of an uncoated tool was higher than that of the coated tools at a feed 0.075 mm/rev and cutting speed 20 m/min. As shown in Figure 11, the tangential forces and radial forces of uncoated tool (K313) increased severely from the feed 0.05 mm/rev to 0.075 mm/rev compared with that of the coated tools. But the cutting temperature of the uncoated tool (K313) did not increase severely from the feed 0.05 mm/rev to 0.075 mm/rev compared with the coated tools in the simulation models. This phenomenon can be explained by that the critical wear of the uncoated tools (K313) at feed 0.075 mm/rev was not considered in the finite element simulation. The results were consistent with the research of Devillez et al. [37].

## 6. Conclusions

The influences of PVD Ti_(1*−x*)_Al*_x_*N coated tools on the cutting temperature in turning Inconel 718 were analyzed with mathematical analysis model and a finite element simulation model. The results were verified with an orthogonal cutting experiment. The cutting forces and the heat generation in the cutting zone were obtained to analyze the influences of PVD Ti_0.55_Al_0.45_N coating and PVD Ti_0.41_Al_0.59_N coating on the cutting temperature. The main conclusions can be drawn as follows:

(1) The grain preferred orientations (111) and (200) of PVD Ti_0.41_Al_0.59_N coating were more evident compared with that of PVD Ti_0.55_Al_0.45_N coating. The epitaxy growth of TiAlN crystals did not exist in PVD Ti_1−*x*_Al*_x_*N coated cemented carbide tool for low deposition temperature. PVD Ti_0.41_Al_0.59_N coating had better crystallinity than PVD Ti_0.55_Al_0.45_N coating.
(2) The pinning effect of coating increased with the increase of Al concentration, which can help to decrease the friction coefficient between cutting tool and Inconel 718 materials. Compared with PVD Ti_0.55_Al_0.45_N coated tools, PVD Ti_0.41_Al_0.59_N coated tools increased the coating hardness, critical loads and thermal conductivity.
(3) Compared with PVD Ti_0.55_Al_0.45_N coated tools, PVD Ti_0.41_Al_0.59_N coated tools increased the maximum temperature of the workpiece and the maximum temperature of the coated tool compared with the uncoated tool. PVD Ti_0.41_Al_0.59_N coated tools decreased the heat generation and the temperature of the tool body to reduce the thermal stresses generated in the tools.
(4) In this experiment, the PVD Ti_0.41_Al_0.59_N and Ti_0.55_Al_0.45_N coated tools used can improve the wear resistance of tools.

## Figures and Tables

**Table 1:** The mechanical properties of PVD Ti_1−*x*_Al*_x_*N coatings at the room temperature.

*x*	HV_0.025_ (GPa)	Critical Loads (N)	Roughness *Ra* (μm)	Friction Coefficient (Dry)
0.45	13.18	31.67 ± 2.37	0.561 ± 0.003	0.35
0.59	15.05	38.27 ± 0.67	0.516 ± 0.023	0.33

**Table 2:** Temperature dependent mechanical and thermo-physical properties of Ti_0.41_Al_0.59_N coating, Ti_0.55_Al_0.45_N coating and tungsten-based cemented carbide.

	Values at the Following Various Temperatures				
	100 °C	300 °C	500 °C	700 °C	900 °C
Properties of Ti_0.41_Al_0.59_N coating [14,27,33]					
Young’s modulus, GPa	370 (assumed as unchanged with temperature)				
Poisson’s ratio	0.22 (assumed as unchanged with temperature)				
Density, kg/m^3^	1892 (assumed as unchanged with temperature)				
Thermal conductivity, W/(m·K)	12.61	14.01	15.41	16.81	18.21
Specific heat, J/(kg·K)	639.89	727.28	769.46	794.29	810.67
Properties of Ti_0.55_Al_0.45_N coating [14,27,33]					
Young’s modulus, GPa	370 (assumed as unchanged with temperature)				
Poisson’s ratio	0.22 (assumed as unchanged with temperature)				
Density, kg/m^3^	1892 (assumed as unchanged with temperature)				
Thermal conductivity, W/(m·K)	10.61	12.01	13.41	14.81	16.21
Specific heat, J/(kg·K)	639.89	727.28	769.46	794.29	810.67
Properties of tungsten-based cemented carbide [27]					
Young’s modulus, GPa	534 (assumed as unchanged with temperature)				
Poisson’s ratio	0.22 (assumed as unchanged with temperature)				
Density, kg/m^3^	11900 (assumed as unchanged with temperature)				
Thermal conductivity, W/(m·K)	40.15	48.55	56.95	65.35	73.75
Specific heat, J/(kg·K)	346.01	370.01	394.01	418.01	442.01

**Table 3:** The special parameters of adiabatic shear fracture in the cutting zone for Ti_0.41_Al_0.59_N coated tool (KC5010), Ti_0.45_Al_0.55_N coated tool (KC5025) and uncoated tool (K313) tools with variations of feed at a cutting speed of 20 m/min [32].

Type	*f* (mm/rev)	φ (°)	L (mm)	B	*q*_shear_ (J/(mm^2^·s))
KC5010	0.025	28.11 ± 0.22	0.0843 ± 0.0105	1.129	67.2738 ± 0.0351
	0.050	31.04 ± 0.15	0.1522 ± 0.0078	0.873	63.3973 ± 0.0530
	0.075	32.23 ± 0.11	0.2203 ± 0.0156	0.751	50.6745 ± 0.0236
KC5025	0.025	26.21 ± 0.19	0.0838 ± 0.0245	1.129	70.0889 ± 0.2371
	0.050	29.13 ± 0.15	0.1538 ± 0.0218	0.873	65.4005 ± 0.1052
	0.075	30.52 ± 0.24	0.1891 ± 0.0027	0.751	54.8375 ± 0.0032
K313	0.025	27.84 ± 0.11	0.0831 ± 0.0178	1.129	66.6349 ± 0.0261
	0.050	29.53 ± 0.21	0.1564 ± 0.0205	0.873	63.4259 ± 0.0331
	0.075	29.21 ± 0.15	0.2143 ± 0.0137	0.751	60.5648 ± 0.0082

## Nomenclature

*B*	Fraction of the shear plane heat conducted into the workpiece material
*q*_shear_	Heat liberation intensity of a moving shear plane heat source, J/(mm^2^·s)
*α*	Thermal diffusivity, m^2^/s
*x, y, z*	Spatial coordinate axis, mm
X,Y,Z	Spatial coordinate axis of machine tool, mm
*l_i_*	Location of the differential small segment of the shear band heat source *dli* relative to the upper end of it and along its width, mm
*t*	Time, s
*V*	Cutting speed, m/min
*f*	Feed, mm/rev
*a_p_*	Depth of cut, mm
*t*_c_	undeformed chip thickness, mm
*w*	Width of shear plane heat source, mm
*L*	Length of shear plane heat source, mm
*F*_t_	Radial force or feed force, N
*F*_c_	Tangential force, N
*K*_0_	Modified Bessel function of second kind of order zero
*T*	Temperature, °C
*T*_max-workpiece_	Maximum temperature of workpiece during cutting process, °C
*u*	Integral variable of shear band length
*T_p_*_rimary_	Temperature generated by the primary shear heat source, °C
*T*_imaginary_	Temperature generated by the imaginary shear heat source, °C
*T*_max-tool_	Maximum temperature of tool during cutting process, °C
*T*_max-substrate_	Maximum temperature of substrate during cutting process, °C
Greek symbols	
*φ*	Shear angle, °
*γ*_0_	Rake angle, °
*λ*	Thermal conductivity, W/(m·K)
*ρ*	Density, kg/m^3^
*ω*	Function of the time variable *t*
*β*	Relief angle, °
